# FOSTERING COMPASSIONATE CAREGIVING USING CNA TRAINING

**DOI:** 10.1093/geroni/igae098.0897

**Published:** 2024-12-31

**Authors:** Samantha Cotton

**Affiliations:** University of Louisville, Louisville, Kentucky, United States

## Abstract

Behavioral health complications in individuals with Alzheimer’s disease and related dementias (ADRD) present significant challenges in long-term care facilities. The progressive decline in cognitive abilities and subsequent difficulty in managing behaviors related to ADRD necessitate a specialized approach for Certified Nursing Assistants (CNAs) who provide daily care. Addressing these challenges, our HRSA funded Geriatric Workforce Enhancement Program (GWEP) at the University of Louisville has introduced a compassionate care framework tailored for CNAs in long-term care settings. This comprehensive training model equips CNAs with essential skills and knowledge to understand and navigate the behavioral complexities of ADRD. The training encompasses online micro-modules for self-paced learning, interactive workshops for hands-on practice, and an innovative iOS app has been created as a readily available resource for CNAs, offering guidance and support for managing challenging behaviors on-the-go. Our training program has successfully educated 175 CNAs, evidenced by significant improvements in knowledge and self-efficacy regarding ADRD care, as measured by a pre-test/post-test design. The GWEP compassionate care initiative has shown promising results in enhancing the capabilities of CNAs in ADRD care. The multi-pronged educational strategy has proven effective in improving behavioral management, indicating a scalable and empowering model for CNAs. This model signifies a progressive shift towards better ADRD care in long-term facilities, ultimately enriching the quality of life for those with ADRD. Further discussions will focus on the broader application of such programs in long-term care environments.

